# Bilateral internal jugular vein thrombosis due to malignant tumor

**DOI:** 10.1186/s13256-017-1556-0

**Published:** 2018-02-20

**Authors:** Laura Leci-Tahiri, Harieta Zherka-Saracini, Afrim Tahiri, Adhurim Koshi

**Affiliations:** 1 Clinic of Vascular Surgery, University Clinical Center of Kosovo, 10000 Pristina, Kosovo; 2 Clinic of General Surgery, University Clinical Center of Kosovo, 10000 Pristina, Kosovo

**Keywords:** Internal Jugular Vein, Malignant tumor, Thrombosis, Treatment

## Abstract

**Background:**

The aim of the study was to analyze characteristics of patients with bilateral internal jugular vein thrombosis in our department during a 1-year period.

Internal jugular vein thrombosis refers to an intraluminal thrombus occurring anywhere from the intracranial internal jugular vein to the junction of the internal jugular vein and the subclavian vein, which form the brachiocephalic vein. It can occur spontaneously or as a complication of head and neck infections, surgery, central venous lines, local malignancy, polycytemia, hyperhomocysteinemia, neck massage, or intravenous drug abuse. Spontaneous bilateral internal jugular vein thrombosis may occur as a result of a neoplasm, a condition called Trousseau’s syndrome.

**Methods:**

The medical records of four patients with internal jugular vein thrombosis were reviewed for patient clinical characteristics, including age, sex, and other diseases. This is a retrospective study, and we analyzed four patients who had distant malignant tumors.

**Results:**

During a 1-year period, four male patients were referred to our department for bilateral internal jugular vein thrombosis. Three of them had lung neoplasm, and one had urinary tract neoplasm. Three patients had thrombosis in the upper arm at the same time, one of them in both arms. Therapy consisted of unfractioned heparin in all patients. The main clinical manifestations were pain and cervical edema. The time between the first clinical manifestation and diagnosis of internal jugular vein thrombosis was 4 days. In the current study, no patient exhibited complications due to internal jugular vein thrombosis.

**Conclusions:**

Diagnosing internal jugular vein thrombosis requires a high degree of suspicion. Our study underlines that bilateral internal jugular vein thrombosis is a risk indicator for malignancy. In our literature review of internal jugular vein thrombosis, 85% of patients exhibited unilateral thrombosis, whereas the remaining patients had bilateral thrombosis (15%). The knowledge of predictive factors of internal jugular vein thrombosis seems to be of utmost importance to improve patient management.

## Background

Jugular vein thrombosis is a serious condition and refers to intraluminal thrombus anywhere from the intracranial jugular vein to the junction between the internal jugular vein (IJV) and subclavian vein, which form the brachiocephalic vein. IJV thrombosis was first described by Long as a complication of peritonsillar abscess in 1912 [1–3].

It may be a secondary thrombosis as a complication of head and neck infections, surgery and trauma, local skin infections, throat infections, local or distant malign tumor (Trousseau’s syndrome), central venous catheter placement, intravenous drug abuse, polycythemia, neck massage, Lemierre’s syndrome, ovarian hyperstimulation syndrome, hypercoagulable state secondary to factor V Leiden, protein C, protein S, and antithrombin III deficiency, or it may be a primary internal jugular vein thrombosis [1, 3–5].

The symptoms may include pain and swelling to the neck (at the angle of the jaw), a palpable cord beneath the sternocleidomastoid, or other clinical manifestations as fever, leukocytosis, sepsis syndrome, etc., and can have life-threatening complications such as pulmonary edema, laryngeal and lower airway swelling, superior sagittal sinus thrombosis, intracranial hypertension, cerebral edema, septic emboli, chylothorax, renal failure, or superior vena cava syndrome [3–7].

Venous duplex ultrasonography is the diagnostic test of choice for many with IJV thrombosis. Ultrasonographic findings include a dilated and incompressible vein, intraluminal clot (a late finding), and no response to the Valsalva maneuver (expected change in intraluminal volume secondary to enhanced venous return) [2, 4, 6, 8].

Contrast-enhanced computed tomography (CT) may be useful for diagnosing suspected IJV thrombosis. CT findings include low-density intraluminal thrombus, a sharply defined bright vessel wall (because of contrast uptake by the vasa vasorum), soft tissue swelling surrounding the IJV, and a distended IJV proximal to the thrombus [2, 5].

Anticoagulants, fibrinolytics, and antibiotic drugs can be used to treat IJV thrombosis. Sometimes, if needed, a superior cava vein filter can be used to prevent pulmonary embolism (PE). Although surgical intervention is rarely necessary, infected thrombophlebithis, associated with a deep neck infection, calls for drainage of any fluid collection and debridement of all infected tissue [4, 5, 7].

## Methods

The medical records of four patients with IJV thrombosis were reviewed for patient clinical characteristics, including age, sex, and other diseases. This is a retrospective study, and we analyzed four patients who had distant malignant tumors. The main symptoms were neck and moderate facial swelling, and pain. The neck physical examination showed bilateral cord-like structures on both sides, extending from the angle of the mandible and beneath the sternocleidomastoid muscles, without neck lymphadenopathy.

We used Doppler ultrasonography to diagnose IJV thrombosis in all patients. Heparin was given intravenously, and antibiotics and analgetics were also administered. The patients were discharged on oral anticoagulants. All patients were followed up after hospital discharge.

## Results

The average age of the patients was 60.5 years old. All of them were Albanian male. They were hospitalized in our clinic for 10 days. All of them had cervical edema, with superficial collateral dilated veins, and indurated bilateral IJV (Table 1). The average time between symptom onset and diagnosis was 4 days.

**Table 1:** Clinical characteristics of patients

Clinical characteristics	No. of patients
Malignant disease	4
Cervical edema	4
Arm edema	3
Physical findings of SVC syndrome	3
Location of associated upper limb deep vein thrombosis:	
Left upper extremity	3
Right upper extremity	0
Bilateral	1
Superficial collateral dilated veins	4
Indurated bilateral IJV	4

*IJV* internal jugular vein, *SVC* superior vena cava

**Fig. 1:**
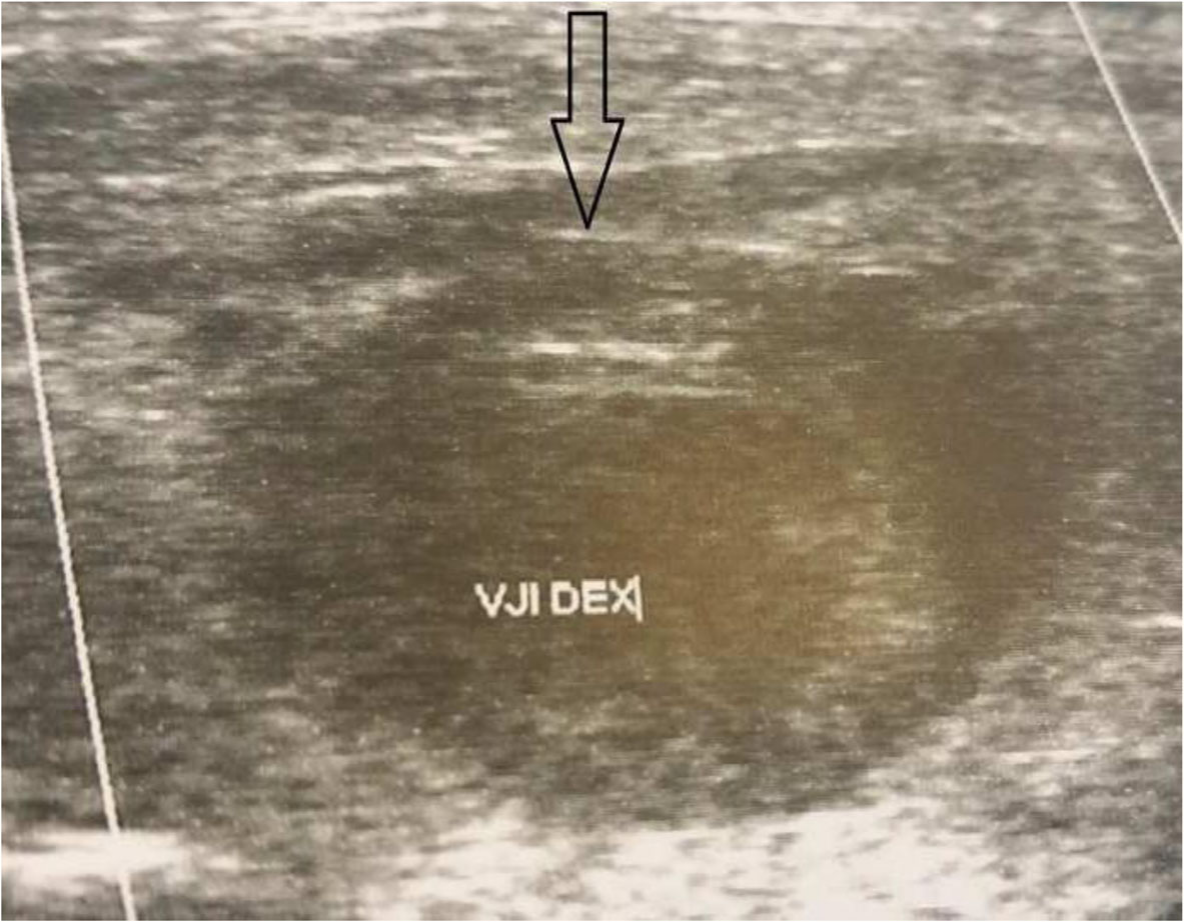
Doppler ultrasound of the right internal jugular vein (arrow) showing filling defect suggestive of thrombosis

They had no history of neck surgery, central venous catheterization, or coagulation disorders. Doppler sonography of the neck performed in the supine position showed bilateral IJV filled with high echogenicity, which could not be collapsed by pressure (Fig. 1).

**Fig. 2:**
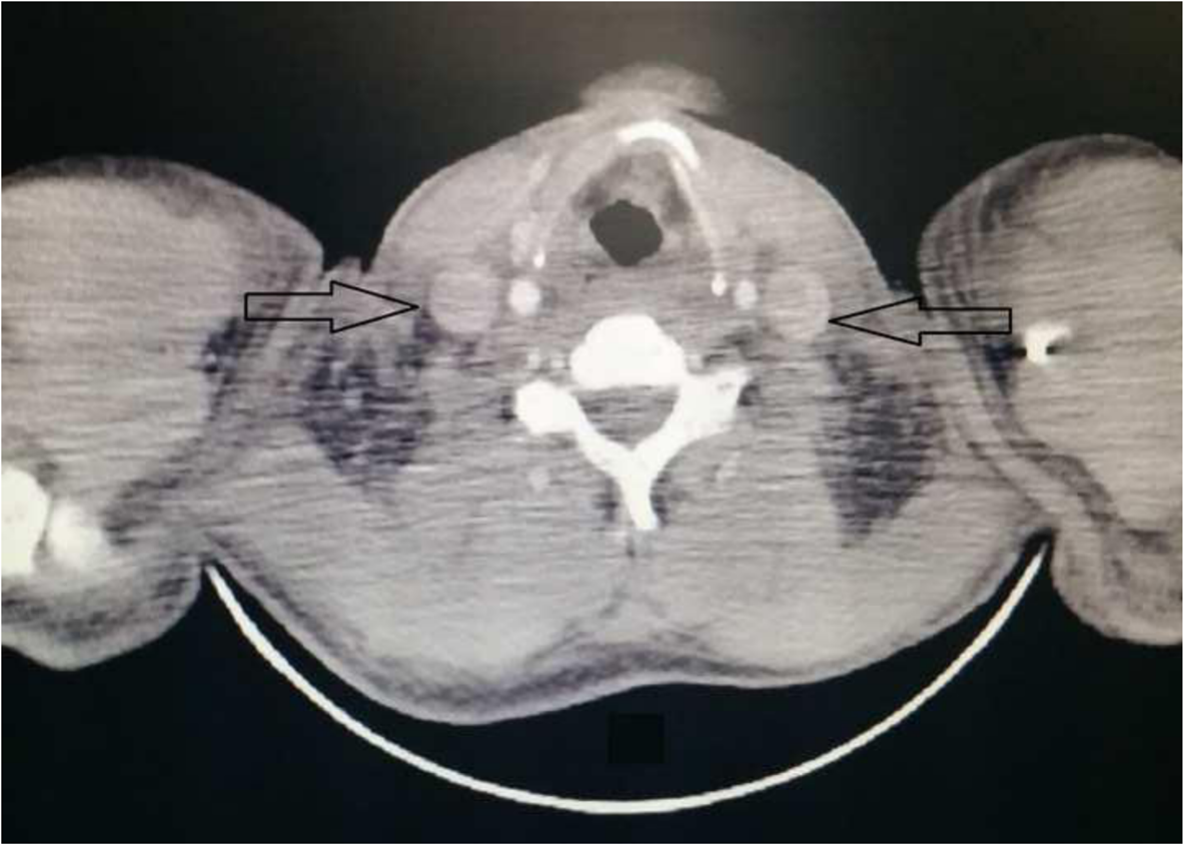
Cervical computed tomography scan shows bilateral internal jugular vein (arrows) thrombosis

A cervical and thoracic CT scan was characterized by low attenuation areas that completely filled the lumen of the IJV bilaterally. There was no mass and mediastinal lymph node (Fig. 2).

Laboratory data were: C-reactive protein (CRP) level: 69, white blood cell (WBC) count: 13,000, and D-dimer level: 400.

At first, they were all treated by a cardiopulmonary disease internist. Patients with associated upper extremity deep vein thrombosis (DVT) underwent concomitant elastic compression stocking of the involved upper extremity.

They all continued treatment under an oncologist and two of them died within a year.

## Discussion

Thrombosis of the IJV is a rare and underdiagnosed condition. Previously reported causes include central venous catheterization, head and neck infections, such as in Lemierre’s syndrome, malignancy, aneurysm, intravenous drug abuse, and idiopathic and iatrogenic injury. Increasing age, obesity, and associated illness have also been attributed as causes. External compression over the vein has also been reported as a possible cause [1, 4, 6–9].

The time to diagnosis of bilateral IJV thrombosis has been found to be variable. Our results are in accordance with results from the literature, the median interval between the first clinical manifestation onset and diagnosis of IJV thrombosis was 4 days [2, 3, 7–10].

Despite the limited sample of patients, our series underlines that bilateral IJV vein thrombosis is a significant risk indicator of malignancy.

The risk of pulmonary emboli is truly unknown. Due to the increased risk of further potentially life-threatening thrombotic episodes, such as the risk of PE, patients with a thrombosed IJV often receive anticoagulation [4, 5, 9, 10].

The most commonly quoted rate of PE occurring in the setting of IJV thrombosis is 5%; however, this statistic is taken from a relatively small retrospective study performed more than 25 years ago.

The morbidity and mortality of internal jugular vein thrombosis are comparable to those of upper extremity DVT; accordingly, consideration should be given to treat these two entities in a similar way. A later retrospective study demonstrated PE rates of 0.5% and 2.4% for isolated IJV thrombosis and combined subclavian/axillary vein and IJV thrombosis, respectively [6–8].

Related malignancies are also exceptional and not well documented in the etiology of IJV thrombosis. In our study, we have noticed that patients with bilateral IJV thrombosis all have malignant disease. Among them, lung cancer appeared most frequently. The relationship between cancer and thrombosis was first described by Jean Baptiste Bouillard in 1823. Later on, the association of cancer and thrombophlebitis was observed by Armand Trousseau, who in 1861 stated that if the diagnosis of a suspected carcinoma of an internal organ could not be verified, the sudden and spontaneous appearance of thrombophlebitis in a large vein afforded necessary proof for diagnosis. He showed the relationship between gastric carcinoma and thrombosis [8–10].

In the setting of infection, many patients do well when given antibiotics alone, without anticoagulant therapy. However, in the presence of septic emboli or with clear evidence of clot propagation, many physicians choose to add systemic anticoagulation, as in our patients [4, 8–10].

## Conclusions

Bilateral IJV thrombosis is extremely rare and can be an indicator of early malignancy. Early diagnosis is very important in preventing complications. In conclusion, thrombosis of the IJV is a rare condition that may progress to upper limb DVT. Several causes of thrombosis of the IJV have previously been reported, including malignant diseases as in our cases.

Treatment of the malignancy is the most definitive therapy, but usually in this particular disease is also unsuccessful, as in our cases.

Our findings underscore that the search for cancer should be made routinely, especially in patients with bilateral IJV thrombosis.

## References

[1] Robbins S, Cotran RS, Kumar V. Pathologic basis of diseases. 3rd ed. Philadelphia: W.B. Saunders Co.; 1984. p. 314–55.

[2] Chen K-H, Chen Y-J, Liaw C-C, Chang H-J, Yeow K-M. Left internal jugular vein thrombosis due to a lung tumor. Chang Gung Med J. 2003;26(6):458–61. 12956295

[3] Gbaguidi X, Janvresse A, Benichou J, Cailleux N, Levesques H, Marie I. Internal jugular vein thrombosis: outcome and risk factors. Q J Med. 2011;104:209–19. 10.1093/qjmed/hcq179 20974769

[4] Hindi Z, Fadel E. Idiopathic bilateral external jugular vein thrombosis. Am J Case Rep. 2015;16:554–7. 10.12659/AJCR.895124 PMC4554332 26301793

[5] Erkoç R, Uzun K, Yuca K, Etlik O, Doğan E, Sayarlıoğlu H, İşlek A, Çankaya H. Internal jugular vein thrombosis trwo different etiologies. Eur J Gen Med. 2005;2(3):123–8.

[6] Bandara A, Wimalarathna H, Kalupahana R, Gunathilake S. Internal jugular venous thrombosis due to Trousseau’s syndrome as the presenting feature of metastatic prostate carcinoma: a case report. J Med Case Reports. 2016;10:104. 10.1186/s13256-016-0884-9 PMC4839162 27097872

[7] Shameem M, Akhtar J, Bhargava R, Ahmed Z, Baneen U, Khan N. Internal jugular vein thrombosis – A rare presentation of mediastinal lymphoma. Respir Med CME. 2010;3:273–5.

[8] Shakeel M, Keh M, Kynaston J, McCluney N, Ah See KW. Evidence based management of spontaneous internal jugular vein thrombosis: a literature review. J Otolaryngol-ENT Res. 2015;2(2):1–7.

[9] Carrington BM, Adams JE. Jugular vein thrombosis associated with distant malignancy. Postgrad Med J. 1988;64:455–8. 10.1136/pgmj.64.752.455 PMC2428885 3211826

[10] Yalaza M, Kafadar M, Civgin E, Duzgun A. External jugular vein thrombosis as a sign of metastatic breast cancer. J Breast Health. 2017;13(1):43–5. 10.5152/tjbh.2016.3315 PMC5351464 28331768

